# Genetic mechanisms underlying local spontaneous brain activity in episodic migraine

**DOI:** 10.3389/fnins.2024.1348591

**Published:** 2024-02-06

**Authors:** Wei Gui, Fengqing Lu, Lulan Fu, Ziru Deng, Xiuxiu Zhao, Wenwen Cheng, Ying Yang, Yu Wang

**Affiliations:** ^1^Department of Neurology, Epilepsy and Headache Group, The First Affiliated Hospital of Anhui Medical University, Hefei, China; ^2^Department of Neurology, The First Affiliated Hospital of USTC, Division of Life Sciences and Medicine, University of Science and Technology of China, Hefei, China; ^3^Department of Neurology, The Second Affiliated Hospital of Anhui Medical University, Hefei, China; ^4^Anhui Provincial Stereotactic Neurosurgical Institute, The First Affiliated Hospital of USTC, Division of Life Sciences and Medicine University of Science and Technology of China, Hefei, China; ^5^Department of Radiology, The First Affiliated Hospital of USTC, Division of Life Sciences and Medicine University of Science and Technology of China, Hefei, China

**Keywords:** migraine, Allen Human Brain Atlas, gene, regional homogeneity, functional MRI

## Abstract

Advances in neuroimaging techniques during the past few decades have captured impaired functional brain activity in migraine disorders, yet the molecular mechanisms accounting for its alterations in migraine remain largely unknown. A total of 27 patients with episodic migraine (EM) and 30 matched healthy controls (HCs) underwent resting-state functional and structural magnetic resonance imaging (MRI) scans. Regional homogeneity (ReHo), low-frequency fluctuations (ALFF), and fractional amplitude of low-frequency fluctuations (fALFF) of fMRI were compared between the two groups. Based on the Allen Human Brain Atlas and risk genes in migraine, we identified gene expression profiles associated with ReHo alterations in EM. Compared with HCs, patients with EM showed increased ReHo in the left orbital part of the superior frontal gyrus (*P* < 0.05, cluster-level FWE-corrected). The expression profiles of 16 genes were significantly correlated with ReHo alterations in EM (*P* < 0.05/5,013, Bonferroni corrected). These genes were mainly enriched for transcription regulation, synaptic transmission, energy metabolism, and migraine disorders. Furthermore, the neural activation was positively correlated with Hamilton Rating Scale for Anxiety (HAMA) scores. To test the stability of our results, we repeated our procedure by using ALFF and fALFF and found these results had a high degree of consistency. Overall, these findings not only demonstrated that regional brain activity was increased in patients with EM, which was associated with emotional regulation but also provided new insights into the genetic mechanisms underlying these changes in migraine.

## Introduction

Current theories of migraine have a strong genetic component with a heritability of 40%−60% (Polderman et al., 2015) and involve activation of complex brain networks (Messina et al., 2022; Yang et al., 2022b). As migraine diagnosed by clinical criteria did not fully capture the heterogeneity and complexity of migraine, detecting migraine-specific biomarkers in genetics, neurophysiology, and structural and functional imaging may provide substantial evidence for improving our understanding of the mechanisms of migraine and identifying new targets for migraine prevention.

Resting-state functional magnetic resonance imaging (fMRI) has been increasingly emerging as a promising technique for measuring spontaneous brain activity based on blood oxygen level-dependent signals of the human brain (Fox and Raichle, 2007; Yang et al., 2022a,b). Numerous studies have consistently shown extensive spontaneous brain activity changes in multiple brain regions and networks in patients with migraine via several approaches, such as amplitude of low-frequency fluctuations (ALFF), fractional amplitude of low-frequency fluctuations (fALFF), and regional homogeneity (ReHo) (Cai et al., 2023). ReHo, a reliable and reproducible data-driven approach in fMRI, measures the degree of functional synchronization between a given voxel and its neighboring voxels (Zang et al., 2004; Yang et al., 2020, 2022a), which has shown high reproducibility and has been repeatedly reported to be changed in migraine (Zhao et al., 2014; Meylakh et al., 2018; Chen et al., 2019). Thus, we were particularly interested in this measure in the current study. However, the genetic mechanisms underlying altered spontaneous brain activity in migraine are largely unknown.

A recent genome-wide analysis of 102,084 migraine cases identified 123 risk loci and subtype-specific risk alleles, which were enriched in both vascular and central nervous system tissue/cell types (Hautakangas et al., 2022). As intermediate phenotypes, neuroimaging features are theoretically closer to the genetic substrates of brain disorders. Therefore, transcription-neuroimaging association studies between brain gene expression and neuroimaging features have been performed in normal people (Chen et al., 2022) and patients with brain disorders (Ji et al., 2021; Xu et al., 2023), which may further explore the genetic mechanisms underlying brain structural and functional abnormalities. The Allen Human Brain Atlas (AHBA; http://human.brain-map.org), which integrates brain-wide gene expression data from six donated human brains, provides a promising avenue to bridge the gap between microscopic molecular function and macroscopic brain alterations (Hawrylycz et al., 2012). However, there has been no transcription-neuroimaging association study to identify genes associated with brain alterations in migraine.

**Figure 1:**
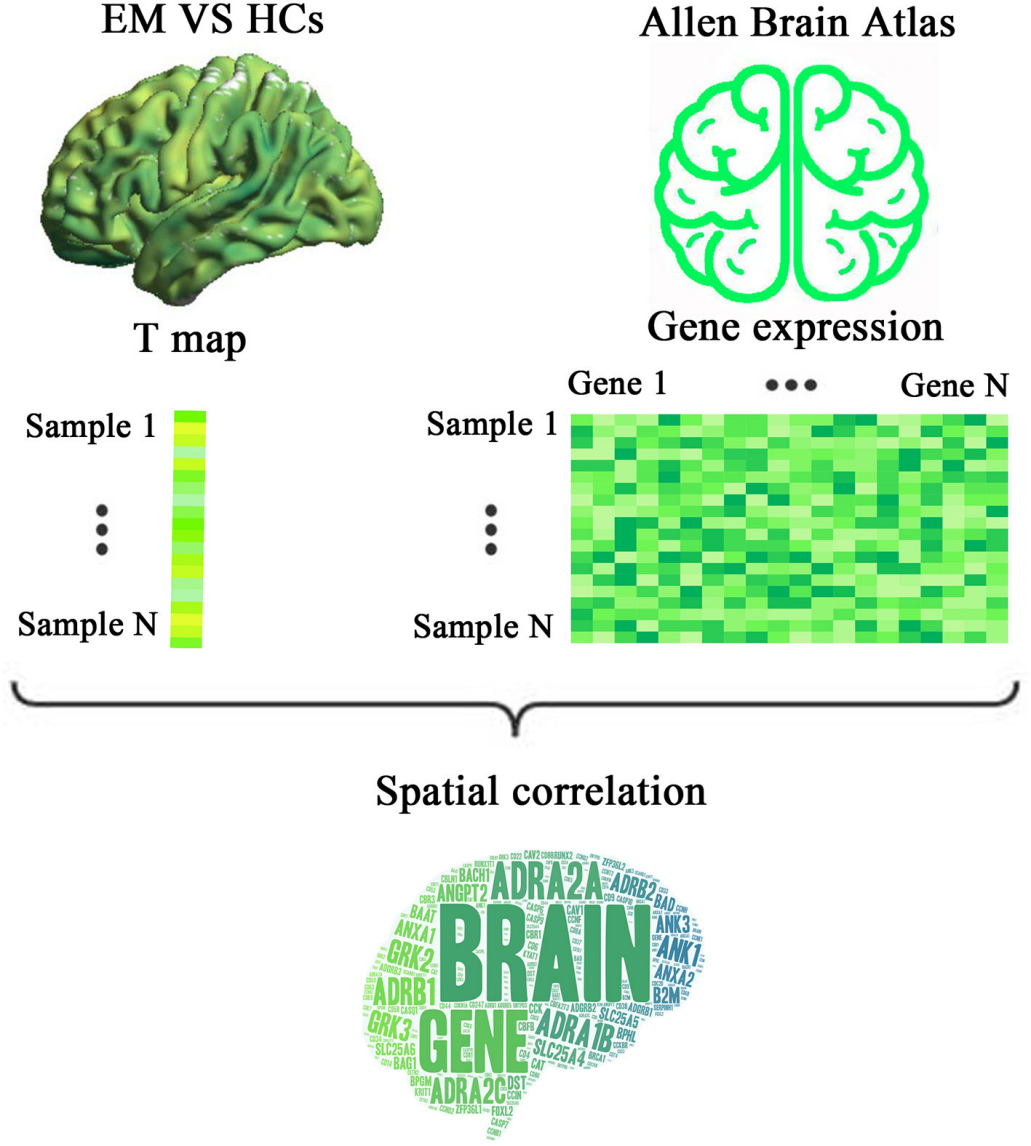
A schematic pipeline of the study design. First, a voxel-wise ReHo difference map was created by comparing the resting state fMRI data of EM patients and HCs. Second, a gene expression matrix was generated from six donated brains from the Allen Human Brain Atlas by using the newly proposed pipeline. Finally, transcriptome-neuroimaging spatial correlation analyses were performed between genes associated with migraine and ReHo changes in EM. ReHo, regional homogeneity; EM, episodic migraine; HCs, healthy controls.

Therefore, we hypothesized that patients with episodic migraine (EM) presented regional brain activity changes compared with HCs, and these differences were related to the expression of migraine risk genes. First, we obtained resting-state fMRI and structural MRI data from 27 EM patients and 30 HCs. A case-control brain activity difference was assessed based on ReHo maps of fMRI. Second, genes associated with ReHo changes in migraine were identified by performing transcriptome-neuroimaging spatial correlation analysis. By using a newly proposed pipeline, the level of gene expression was obtained from the donated AHBA brains. Finally, functional features of the identified ReHo-related genes were analyzed by functional annotation. A schematic pipeline of the study design is shown in Figure 1.

## Methods

### Participants

To achieve a desired power of 90% with a significance level of 5%, the required sample size was 26 subjects for each group, as calculated by G^*^power software (https://www.psychologie.hhu.de/). Between November 2021 and April 2022, a total of 66 right-handed individuals were enrolled in the present study, including 33 patients with EM recruited from the Headache Clinic of the First Affiliated Hospital of the University of Science and Technology of China (USTC; Anhui Provincial Hospital) and 33 age-, sex-, and education-matched HCs recruited from the same sociodemographic environment via poster advertisements. The subjects were enrolled in our study according to the inclusion time. After an MRI scan, two patients had a lacunar cerebral infarction, and four patients had paranasal sinusitis or a cyst. Among HCs, two subjects had paranasal sinusitis, and one subject had tympanitis. Finally, a total of 57 subjects (27 patients with EM and 30 HCs) were enrolled in the present study. Patients with EM were diagnosed according to the International Classification of Headache Disorders-III (ICHD-III). No migraine-preventive medication was used by the participants in the past 3 months. The inclusion criteria for patients and controls included 18–55 years of age, right-handedness, and Han ethnicity. The exclusion criteria were as follows: (I) the presence of other neurological diseases; (II) a history of significant physical or psychiatric illnesses; (III) a history of head injury with loss of consciousness; (IV) any alcohol and drug abuse; and (V) pregnancy or any contraindications for MRI. To avoid measuring imaging changes associated with acute migraine symptoms, all patients were scanned at intervals of at least 72 h after and 24 h prior to a migraine event. The study procedures were approved by the Ethical Committee of the First Affiliated Hospital of USTC and complied with the Declaration of Helsinki. Written informed consent was obtained from all participants before study entry.

### Clinical assessment

Demographic information of the participants (including age, sex, and years of education) was recorded. Migraine family history, migraine duration, the Headache Impact Test-6 (HIT-6) (Yang et al., 2011), and a visual analog scale (VAS) (Gallagher et al., 2001) were used to assess the impact of migraine. The 14-item Hamilton Rating Scale for Anxiety (HAMA) and the Beck Depression Inventory, 2nd edition (BDI-II), were applied to assess the anxiety and depression status of the patients. The Montreal Cognitive Assessment (MoCA) was used to evaluate cognitive function. The Big Five Inventory-60 items (BFI) measure personality traits. Several subjects did not undergo the whole clinical assessment due to personal reasons, e.g., low education level or insufficient time.

### MRI acquisition

All the subjects underwent MRI scans on a GE 3.0T MR system (DISCOVERY MR750, GE Healthcare, Milwaukee, WI, United States) with a 24-channel head coil at the MRI Center of the First Affiliated Hospital of the University of USTC. High-resolution, three-dimensional (3D), T1-weighted structural images were acquired using a brain volume (BRAVO) sequence with the following parameters: repetition time (TR) = 8.5 ms; echo time (TE) = 3.2 ms; flip angle (FA) = 12°; field of view (FOV) = 256 mm × 256 mm; matrix = 256 × 256; slice thickness =1 mm, no gap; 144 axial slices; and acquisition time = 240 s. Resting-state BOLD data were acquired using a gradient-echo single-shot echo planar imaging (GRE-SS-EPI) sequence with the following parameters: TR = 2,000 ms; TE = 30 ms; FA = 90°; FOV = 240 mm × 240 mm; matrix = 64 × 64; slice thickness = 4 mm without gap; 36 interleaved axial slices; 240 volumes; and acquisition time = 480 s. In addition, conventional MRI examinations were conducted to exclude subjects with cerebral infarction, malacia, or occupying lesions.

### fMRI data preprocessing

The functional data were preprocessed and analyzed using the Statistical Parametric Mapping software (SPM12; http://www.fil.ion.ucl.ac.uk/spm) and the Data Processing and Analysis for Brain Imaging (DPABI_ V3.1_180801; http://rfmri.org/dpabi) (Yan et al., 2016) in MATLAB R2016b (MathWorks, Inc.) as follows: (1) removal of the first 10 volumes of the resting-state functional images; (2) slice timing correction; (3) head motion correction; (4) regression of several nuisance covariates (linear drift, estimated motion parameters based on the Friston-24 model, spike volumes with frame-wise displacement (FD) > 0.5, white matter signal, and cerebrospinal fluid signal) from the data; (5) data detrending and bandpass-filtering from 0.01 to 0.1 Hz; and (6) spatial normalization using the diffeomorphic anatomical registration through the exponentiated Lie algebra (DARTEL) technique. We also calculated FD, which indexes the volume-to-volume changes in head position.

### ReHo, ALFF, and fALFF analyses

The ReHo, ALFF, and fALFF analyses were processed using the DPABI software (V3.1_180801). ReHo can be used to measure the degree of local and regional neural activity coherence. In short, it was calculated as Kendall's coefficient of concordance (or Kendall's W) of the time course of a given voxel with those of its nearest neighbors (26 voxels). ALFF was computed as the mean power spectrum in a specific low-frequency band (0.01–0.1 Hz). fALFF was defined as the ratio of the power spectrum in the low-frequency band (0.01–0.1 Hz) to that in the entire frequency range. For the purpose of standardization, the ReHo, ALFF, and fALFF values of each voxel were divided by the global mean value (mReHo, mALFF, and mfALFF). Finally, the resulting images were spatially smoothed with a 6-mm FWHM Gaussian kernel.

### Brain gene expression data processing

Publicly available brain gene expression data were obtained from the AHBA dataset (http://www.brain-map.org), which was derived from six human postmortem donors (Hawrylycz et al., 2012) (Supplementary Table S1). This dataset consists of normalized microarray expression data for more than 20,000 genes measured in 3,702 brain samples. A new proposed pipeline was used to process gene expression data (Arnatkeviciute et al., 2019). Specifically, probe-to-gene annotations were first updated based on the latest available information from the National Center for Biotechnology Information (NCBI) using the Re-Annotator package. With intensity-based filtering, we excluded probes that did not exceed the background noise in at least 50% of samples across all donors. Since multiple probes were used to measure the expression level of a single gene, we further used the RNA-seq data as a reference to select probes. After excluding genes that do not overlap between RNA-seq and microarray datasets, we computed the correlations between microarray and RNA-seq expression measures for the remaining genes. After excluding probes with low correlations (*r* < 0.2), a representative probe was selected for a gene based on the highest correlation with the RNA-seq data. In this study, we only included tissue samples from the left cerebral cortex. For one, all six donors had expression data in the left hemisphere, but only two donors had samples in the right hemisphere. For another, the inclusion of subcortical samples might introduce potential biases, given the substantial divergence in gene expression between cortical and subcortical regions. To account for potential between-sample differences and donor-specific effects in gene expression, we conducted both within-sample cross-gene and within-gene cross-sample normalization with the scaled robust sigmoid normalization method. Differential stability (DS) is a measure of consistent regional variation across donor brains. Earlier work has reported that genes with high DS demonstrate more consistent spatial expression patterns between donors. Since gene expression conservation across subjects is a prerequisite for the transcriptome-neuroimaging spatial correlations, only genes with relatively more conserved expression patterns were selected for analysis. To achieve this goal, we ranked the genes by their DS values and chose the genes with the top 50% highest DS for the main analysis. After these processing procedures, we obtained normalized expression data for 5,013 genes in 1,280 tissue samples. To ensure reliability, we further restricted our analyses to the samples within a cerebral gray matter mask, resulting in a final sample × gene matrix of 845 × 5,013.

### Identifying genes associated with ReHo alterations in EM

To define the ReHo change of a given brain tissue sample, we defined a 3-mm radius sphere centered at the MNI coordinate of each tissue sample and extracted the mean value from the uncorrected *t*-statistical map of the ReHo difference. Then, gene-wise cross-sample Pearson's correlations between gene expression and *t* values were performed to determine genes whose expression levels were correlated with ReHo alterations. Multiple comparisons were corrected using the Bonferroni method (correction for the number of genes, *P* < 0.05/5,013 = 9.974 × 10^−6^). Based on a recent genome-wide analysis in which 123 migraine risk genes were identified, we selected migraine risk genes correlated with ReHo alterations for further analysis.

### Functional annotation

Those migraine risk genes associated with ReHo alterations in EM were functionally annotated using the ToppGene platform (https://toppgene.cchmc.org/) (Chen et al., 2009). Gene ontology (GO) was used to determine their biological functions, including molecular functions (MFs), biological processes (BPs), and cellular components (CCs). The disease databases were used to determine the related diseases. Moreover, protein-protein interactions (PPIs) analysis was performed using STRING v11.5 (https://string-db.org/) to construct PPIs networks with a medium confidence value of 0.4.

### Statistical analyses

First, the Shapiro-Wilk test was performed to assess data normality. Then, demographic, clinical, and MRI parameters were compared between the EM and HC groups using a two-sample *t*-test or a Mann–Whitney *U-*test. Group differences in gender were tested using the Pearson chi-square test. These statistical analyses were performed using the SPSS 23.0 software package (SPSS, Chicago, β). Voxel-based comparisons of ReHo, ALFF, and fALFF between the EM and HC groups were conducted using the parametric two-sample *t*-tests in the SPM12 software. Multiple comparisons were corrected using a cluster-level family-wise error (FWE) method, resulting in a cluster-defining threshold of *P* = 0.001 and a corrected cluster significance of *P* < 0.05. If a measure exhibited a significant between-group difference in a cluster, the mean value within this cluster was extracted for subsequent region of interest (ROI)-based analysis.

To further detect whether the intrinsic brain activity of the ROIs correlated with clinical variables (e.g., duration of illness, HIT-6, VAS, HAMA, BDI-II, BFI, and MoCA), Pearson or Spearman correlation analyses were conducted. For these correlation analyses, a significant uncorrected threshold was set at *P* < 0.05. Multiple comparisons were corrected using the Bonferroni method with a significance threshold of *P* < 0.05/7 = 0.007 (7 different clinical variables).

### Sensitivity analysis

First, some studies have reported altered ALFF and fALFF in migraine, which also reflected local brain activity. To exclude the effect of similar analytic methods, we repeated analytic procedures to verify the stability of our results. Second, the migraine patients had different clinical parameters, which may affect brain resting-state activity. To test the possible effects of these on our results, we added clinical parameters as nuisance covariates in the ROI-based general linear model.

## Results

### Demographic, clinical, and MRI characteristics

The demographic, clinical, and MRI data of all the subjects are summarized in Table 1. The patients with EM and HCs did not show significant differences in terms of age, sex, education, or BMI. Moreover, the FD and TIV, including GM, WM, and CSF, of both groups did not show any significant differences. With regard to clinical assessment, migraine patients showed significantly higher HAMA scores.

**Table 1:** Demographic, clinical, and MRI characteristics of patients with EM and HCs.

	**EM**	**HC**	***P*-value**
**Demographics**			
N	27	30	
Age	32.00 (31.00, 40.00)	30.50 (26.00, 50.75)	0.37
Female/male	25/2	26/4	0.47
Education level	15.00 (9.00, 16.00)	16.00 (13.50, 17.00)	0.06
BMI	22.13 ± 3.09 (14)	22.31 ± 2.90 (11)	0.82
**MRI characteristics**			
FD (mm)	0.10 (0.07, 0.26)	0.13 (0.08, 0.16)	0.81
TIV (cm^3^)	1,399.87 ± 111.56	1,426.48 ± 135.39	0.43
GM (cm^3^)	606.42 ± 45.64	618.10 ± 50.84	0.37
WM (cm^3^)	478.50 ± 44.96	499.85 ± 54.90	0.12
CSF (cm^3^)	310.67 (271.10, 347.50)	296.68 (264.76, 334.12)	0.51
**Migraine characteristics**			
Family history	17/27	N/A	
Disease duration (years)	9.00 (4.00, 16.00)	N/A	
Aura	7	N/A	
VAS scale	6.37 ± 1.80	N/A	
HIT-6 scale	62.50 (56.50, 64.75)	N/A	
HAMA scale	9.00 (6.50, 20.50)	2.00 (1.00, 6.50)	**<** 0.001^*^
BDI-II scale	6.00 (3.00, 13.00)	3.00 (0.00, 7.00)	0.08
MoCA scale	27.00 (24.25, 28.00)	N/A	
**BFI scale**			
Neuroticism	32.71 ± 6.66	30.86 ± 7.90	0.34
Extraversion	38.00 (33.00, 39.00)	40.0 (37.00, 42.50)	0.06
Openness	36.63 ± 7.76	38.29 ± 5.76	0.43
Agreeableness	43.04 ± 3.87	44.10 ± 3.29	0.33
Conscientiousness	42.46 ± 7.034	44.33 ± 4.00	0.27

Data are shown as the mean ± SD (range) or percentiles 50% (25%, 75%). EM, episodic migraine; HCs, healthy controls; BMI, body mass index; FD, framewise displacement; TIV, total intracranial volume; GM, gray matter; WM, white matter; CSF, cerebrospinal fluid; VAS, Visual Analog Scale; HIT-6, Headache Impact Test; HAMA, Hamilton Rating Scale for Anxiety; BDI-II, Beck Depression Inventory-II; MoCA, Montreal Cognitive Assessment; BFI, Big Five Inventory. ^*^P < 0.05.

### ReHo alterations of EM

**Figure 2:**
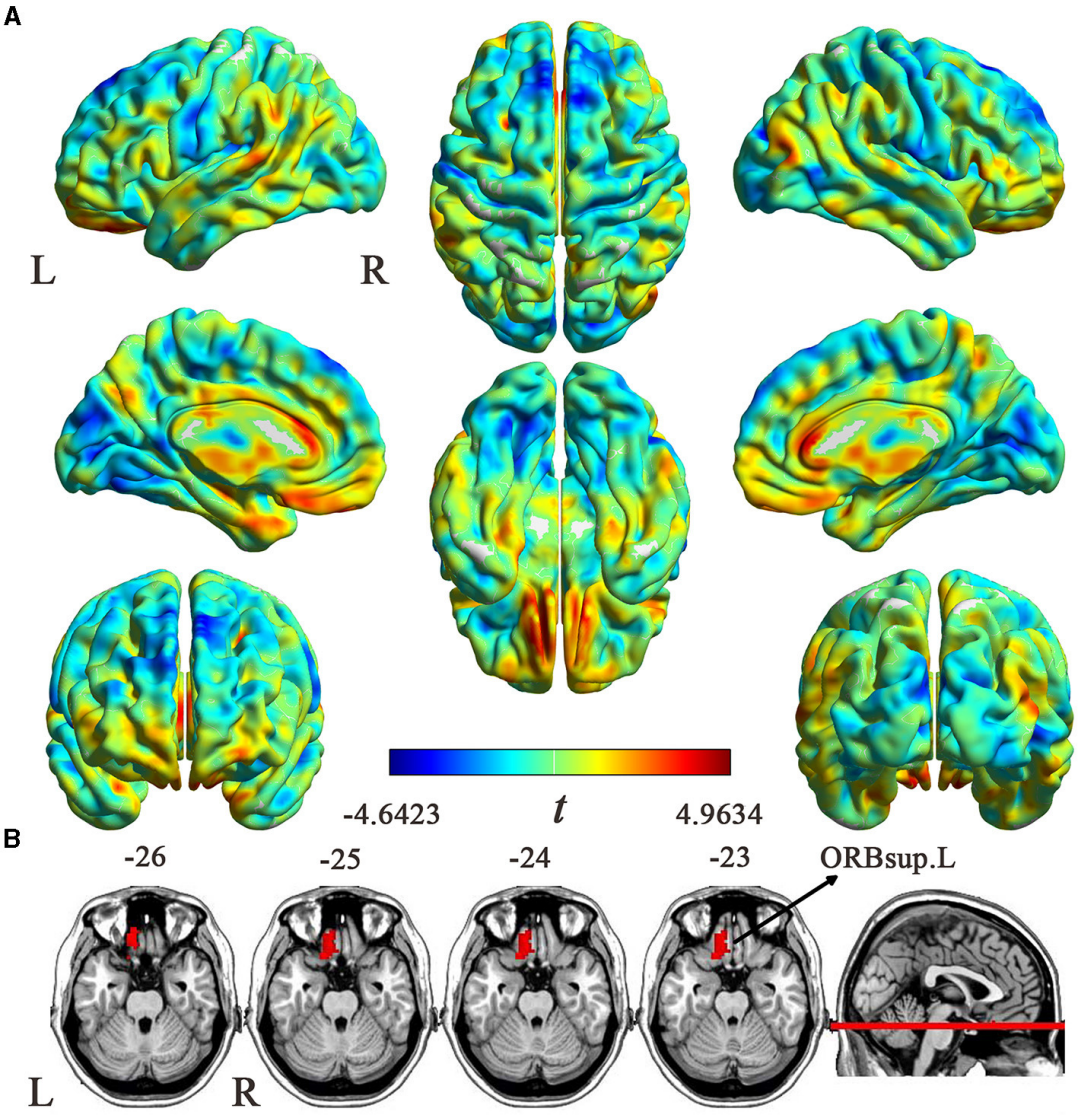
ReHo differences between the EM and HC groups. **(A)** An uncorrected *t* map showing brain voxels with ReHo differences between the two groups. The color bar represents *t*-values. **(B)** Brain region with significantly increased ReHo values (*P* < 0.05, cluster-level FWE corrected) from voxel-wise comparisons between the EM and HCs groups. ReHo, regional homogeneity; EM, episodic migraine; HCs, healthy controls; ORBsup.L, the left orbital part of superior frontal gyrus; L, left; R, right.

The uncorrected case-control ReHo difference map (*t*-statistic map) is shown in Figure 2A. Compared with HCs, patients with EM showed increased ReHo in the left orbital part of the superior frontal gyrus (ORBsup.L) (cluster size = 58 voxels; peak MNI coordinates: x/y/z = −9/36/-27; peak *t* = 4.2551; Cohen's *d* = 0.754) (*P* < 0.05, cluster-level FWE-corrected) (Figure 2B). However, no brain regions showed a significant ReHo decrease in patients with EM.

### Genes related to ReHo alterations in EM

**Figure 3:**
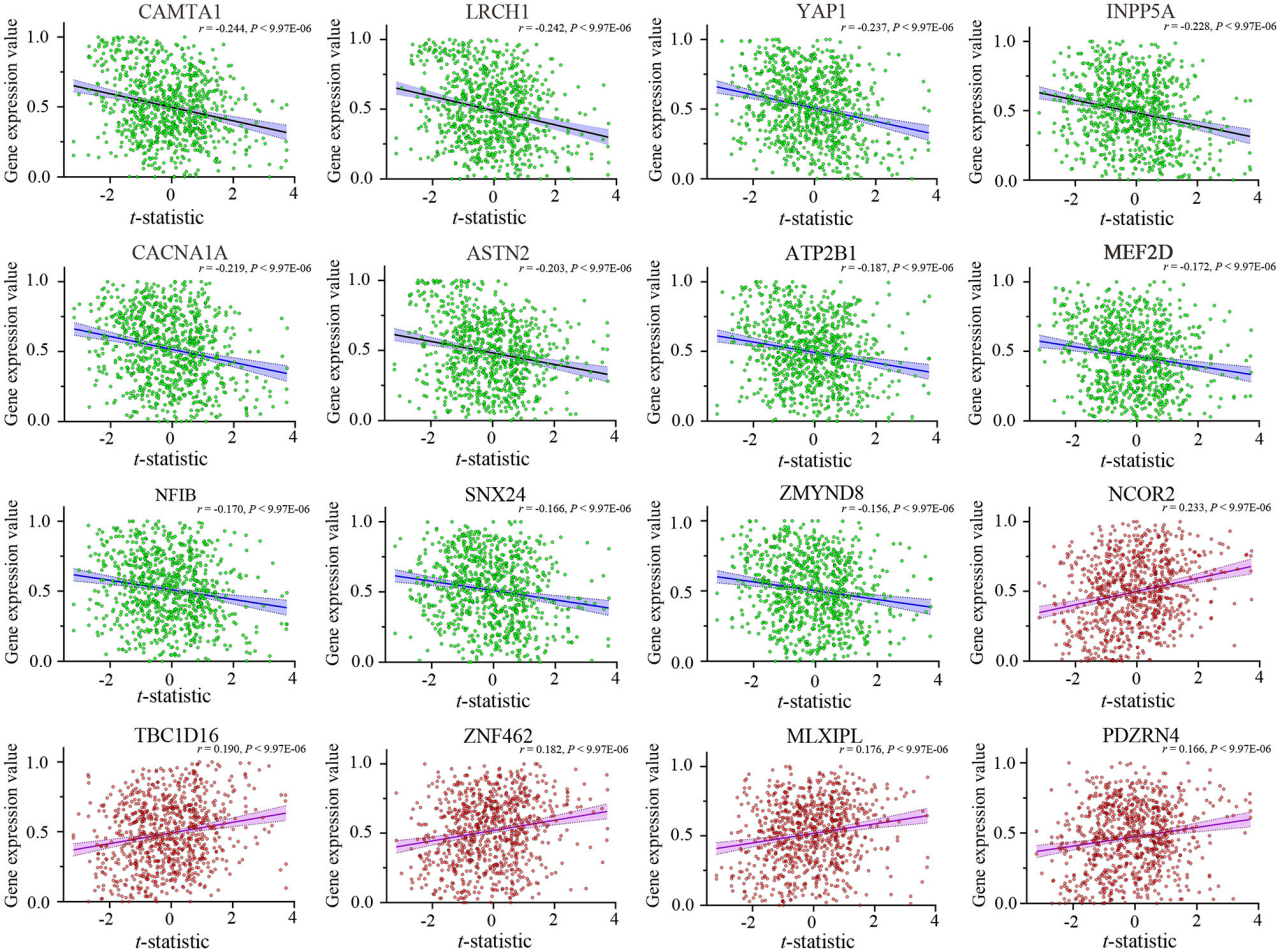
Cross-sample (*n* = 845) correlations between expression values of the 16 genes and ReHo alterations in EM. The x-axis is the *t*-statistic of the ReHo difference between EM and HCs, and the y-axis is the gene expression value. The name of each gene is displayed at the top of each scatterplot, and the correlation coefficient and *P*-value are shown in the upper right corner. ReHo, regional homogeneity; EM, episodic migraine; HCs, healthy controls.

After Brain Gene Expression Data Processing, 5,013 genes for each of the 845 samples were obtained from the AHBA data. A sample-wise spatial correlation was performed between gene expression and ReHo changes in migraine. We found 3,257 genes were significantly correlated with ReHo changes in migraine (*P* < 0.05/5,013 = 9.974 × 10^−6^). Then, only genes with significant correlations among the 123 migraine risk genes were defined as the final target genes. Finally, there were 16 genes that not only showed significant correlations with ReHo changes but also among the 123 migraine risk genes (Figure 3). Here, the negative correlation represented that brain samples with greater ReHo increment in EM showed lower gene expression, and the positive correlation represented that brain samples with greater ReHo increment in EM showed greater gene expression.

### Enrichment analysis

**Figure 4:**
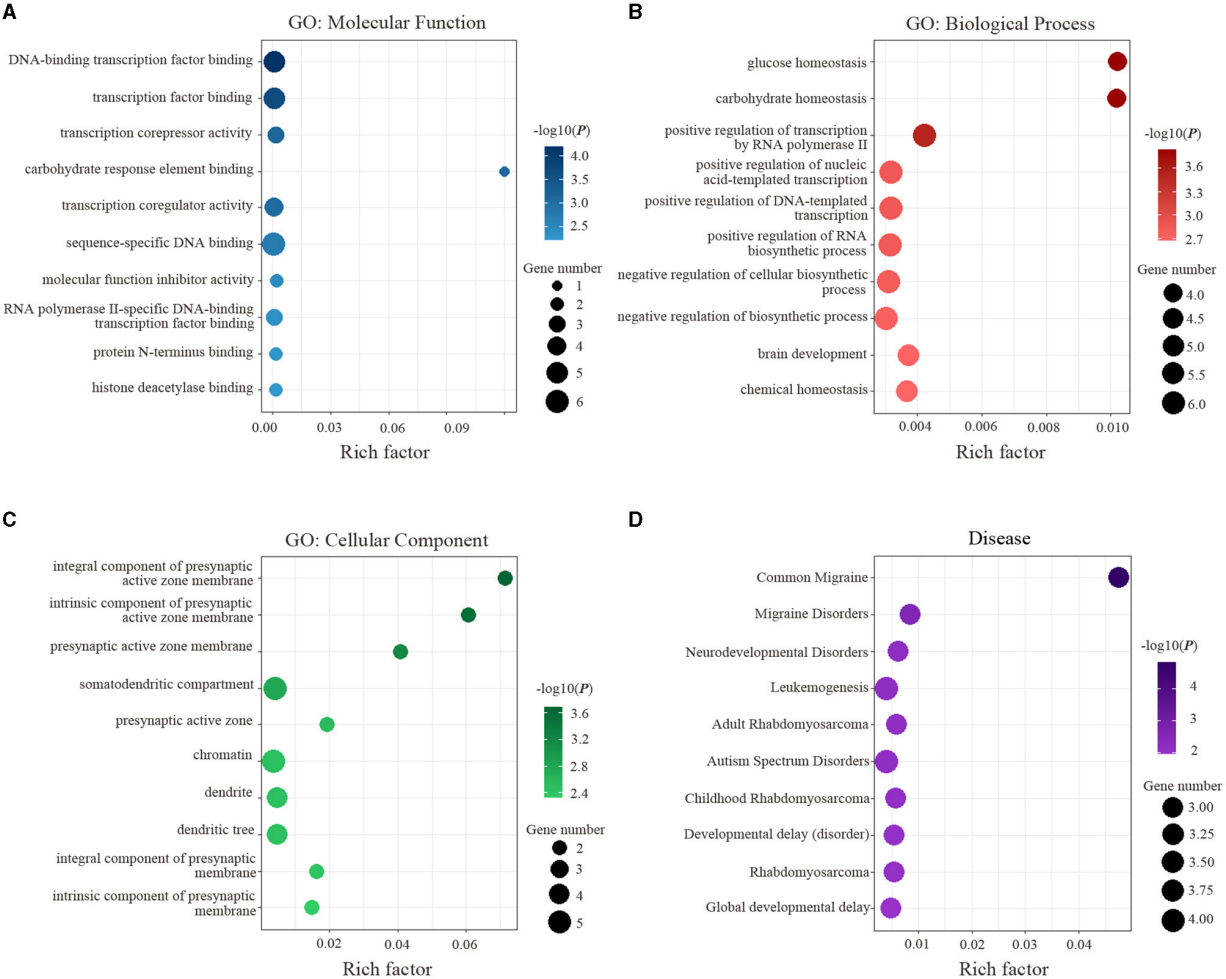
Functional enrichment of the identified 16 genes is significantly associated with ReHo alterations in migraine. **(A)** Significant GO items of molecular function. **(B)** Significant GO items of biological process**. (C)** Significant GO items of a cellular component. **(D)** Significant disease. GO, gene ontology.

The results of enrichment analyses are exhibited in Figure 4. Specifically, the genes related to ReHo alterations of migraine were mainly enriched for MFs. including DNA-binding transcription factor binding (*P* = 6.43 × 10^−5^) and transcription factor binding (*P* = 2.15 × 10^−4^) (Figure 4A); for BPs, including glucose homeostasis (*P* = 1.45 × 10^−4^) and carbohydrate homeostasis (*P* = 1.46 × 10^−4^) (Figure 4B); for CCs, including an integral component of presynaptic active zone membrane (*P* = 2.05 × 10^−4^) and intrinsic component of presynaptic active zone membrane (*P* = 2.85 × 10^−4^) (Figure 4C); for diseases, including common migraine (*P* = 1.45 × 10^−5^) and migraine disorders (*P* = 2.34 × 10^−3^) (Figure 4D).

### PPIs network analysis

Based on 16 genes that showed significant correlations with ReHo alterations and were included in the migraine risk genes, we performed the PPIs network analysis. However, our network did not have significantly more interactions than expected (number of nodes: 16, number of edges: 2, expected number of edges: 1, PPI enrichment *p*-value: 0.146).

### Correlation analyses between MRI data and clinical scales

Further ROI-based correlation analyses revealed that there were significantly positive correlations between ReHo alterations in ORBsup.L and HAMA scores (*rho* = 0.310, *P* = 0.028) in all subjects. However, we did not observe any significant correlation between ReHo alterations and other clinical scales. There were no significant differences between ReHo alterations in ORBsup.L and clinical scales (*P* > 0.007, Bonferroni corrected).

### Sensitivity analysis

First, we repeated our study approach by using ALFF and fALFF. The results were highly consistent with those by ReHo. The results of these sensitivity analyses are presented in detail in the Supplementary Tables S2–S4. Second, to exclude the anxiety severity effect on brain function in patients with EM, we repeated the ROI-based inter-group comparison in ReHo by using a general linear model with HAMA scores, educational level, and age as nuisance covariates. The results showed that the inter-group difference in ReHo of the ORBsup.L was still significant after adjusting for the anxiety severity (F = 9.684, *P* = 0.003), the educational years (F = 15.794, *P* < 0.001), and age (F = 17.759, *P* < 0.001).

## Discussion

To the best of our knowledge, this is the first attempt to identify the associated gene expression with the case-control ReHo difference in patients with episodic migraine. First, increased ReHo was found in the left superior frontal gyrus and orbital part in patients with EM compared with HCs. Second, transcription-neuroimaging analysis identified 16 migraine risk genes whose expression levels were to coincide with ReHo alteration. Third, enrichment analyses showed that these ReHo-related genes were mainly enriched for biological functions including transcription regulation, synaptic transmission, energy metabolism, and migraine disorders. These findings may shed new light on the molecular mechanisms associated with ReHo changes in migraine.

In the present study, we observed that patients with episodic migraine showed significantly increased ReHo in ORBsup.L. The prefrontal cortex (PFC) is involved in the processing of cognitive impairments and is considered a hub of the pain modulatory system (Lorenz et al., 2003; Schmitz et al., 2008). PFC-limic systems, consisting of the PFC, ACC, amygdala and hippocampus, insular cortex, and thalamus, were thought to be associated with emotion and cognition aspects of pain (Talbot et al., 1991; Peyron et al., 2000). In line with our results, existing functional studies have discovered that PFC, including the middle frontal gyrus (MFG), orbital part of the middle frontal gyrus (ORBmid)/superior frontal gyrus (ORBsup)/inferior frontal gyrus (ORBinf), and triangular part of the inferior frontal gyrus (IFGtriang), showed significantly increased local brain activity in patients with migraine (Cai et al., 2023). Thus, taking previous and our findings together, increased ReHo in the PFC may be related to PFC-limic pathway dysfunction, thus contributing to the impaired emotion and cognitive processing of pain in migraine.

It is also interesting to note that increased ReHo values in ORBsup.L were positively correlated with HAMA scores, suggesting that PFC dysfunction may be related to emotional aspects of pain. However, the correlation with the anxiety scores was significant at the uncorrected threshold but did not survive the multiple testing correction. This reflected the trend that higher ReHo changes in ORBsup.L represented more anxiety. To sum up, higher ReHo changes in ORBsup.L may be considered a neuroimaging biomarker to identify EM patients and predict their clinical manifestation.

Transcription-neuroimaging spatial association analysis is a promising avenue for exploring the genetic basis of various neuroimaging phenotypes in both healthy and diseased brains (Zhang et al., 2022). By projecting gene expression data from postmortem human brains and neuroimaging data from living human brains to the same standard space, spatial correlation analysis between gene expression and neuroimaging measurement across brain regions or tissue samples can identify genes associated with neuroimaging phenotypes (Fornito et al., 2019). Previous studies have indicated that the ReHo of spontaneous brain activity may be modulated by neuron-related genes with different functions (Shen et al., 2021). Thus, in the current study, we performed transcription-neuroimaging association analysis to elucidate the genetic mechanisms underlying ReHo alterations in EM. On the basis of a recently published GWAS study, we found 16 migraine-related genes that were associated with ReHo changes between EM and HCs. Hence, it indicated that EM may develop via the joint effects of many migraine-related genes due to the complex nature of this disease. In these 16 genes, most of their expression levels (11 genes) were negative, with *t*-statistic values of ReHo changes in migraine. Negative correlations between gene expression levels and ReHo differences indicated that brain regions with a greater ReHo increment in migraine showed lower gene expression, and positive correlations indicated that brain regions with a greater ReHo increment in migraine showed higher gene expression. For example, we found a negative correlation between MEF2D and the case-control ReHo difference. MEF2D, identified by GWAS hits for common migraine, has been linked to glutamatergic neurotransmission and neuron and synapse development (Tolner et al., 2015). The lower expression level of MEF2D in brain regions with more ReHo increment in migraine may support the hypothesis that abnormalities in brain glucose metabolism generate a mismatch between the brain's energy reserve and metabolic expenditure (Del Moro et al., 2022), which may lead to ReHo increment in migraine. In contrast, NCOR2 showed a positive correlation with the ReHo difference. This gene was higher in brain regions with a higher ReHo increment. NCOR2 mediates the transcriptional repression activity of some nuclear receptors by promoting chromatin condensation, thus preventing access to basal transcription (Lee et al., 2022) and is a novel candidate gene for the migraine-epilepsy phenotype (Nuottamo et al., 2022). The higher expression level of NCOR2 may lead to a ReHo increment by having a more inhibitory effect on gene transcription in migraine.

Enrichment analyses showed that these genes were mainly enriched for transcription regulation, synaptic transmission, physiological homeostasis, and migraine disorders. In the current study, several genes enriched for neurons were mainly involved in aspects of gene transcription regulation, indicating that migraine pathophysiology is associated with multiple transcription factors, i.e., female hormone receptors and receptors for the stress hormone cortisol (MacGregor, 2004). Several genes are enriched for neurotransmission function and neuron and synapse development, which is consistent with the finding that enhancement of synaptic transmission is the neural basis of central sensitization in trigeminovascular neurons of migraine (Latremoliere and Woolf, 2009). Moreover, in terms of genes associated with glucose homeostasis and carbohydrate homeostasis. Increasing evidence has reported brain mitochondrial dysfunction, impaired brain glucose metabolism, and gray matter volume reduction in specific brain areas of migraineurs, suggesting that cerebral metabolic abnormalities might be implicated in migraine pathophysiology (Del Moro et al., 2022).

Protein-protein interactions (PPIs) are a key component of the subcellular molecular networks that enable cells to function (Szklarczyk et al., 2021). In this study, our network does not have significantly more interactions than expected. This means that the current set of proteins is either rather small (i.e., >5 proteins or so) or that it is essentially a random collection of proteins that are not very well connected.

In the sensitivity analysis, we repeated our procedure using the ALFF and fALFF methods. Brain areas with significant differences in the ALFF and fALFF values between EM patients and HCs largely overlapped with ReHo values. Moreover, the correlated gene species and values with resting-state function had a high degree of consistency.

Several limitations should be mentioned in the present study. First, transcriptional data were from the six non-Asian AHBA postmortem brains, whereas neuroimaging data were obtained from 57 Asian living participants. On account of the large gene expression variation across individuals, this discrepancy might lead to uncertain results in the transcription-neuroimaging association study. Second, our gene expression data was limited to the left cerebral cortex and did not include the right hemisphere and subcortical regions, which may introduce potential biases. Finally, the relatively small sample size, no classification of EM, and lack of some clinical assessments may limit the statistical power of detecting brain functional alterations and uncovering potential gene-brain-behavior relationships. Therefore, a larger sample of patients with migraines and more complete clinical data should be included in future studies to validate our findings.

## Conclusion

Increased spontaneous regional brain activity in the left orbital part of the superior frontal gyrus was found in patients with episodic migraine. Furthermore, 16 migraine-related genes showed significant correlations between gene expression and ReHo changes, which were mainly enriched for transcription regulation, synaptic transmission, energy metabolism, and migraine disorders. These findings may help to better understand the genetic mechanisms underlying brain functional abnormalities in migraine and provide new targets for migraine prevention. In the future, studies using physiological biomarkers (regulation of gene expression) in episodic migraine should be performed to substantiate this result.

## Data availability statement

The raw data supporting the conclusions of this article will be made available by the authors, without undue reservation.

## Ethics statement

The studies involving humans were approved by the study procedures were approved by the Ethical Committee of The First Affliated Hospital of USTC (YD2070002011). Written informed consent was obtained from all participants before study entry. The studies were conducted in accordance with the local legislation and institutional requirements. The participants provided their written informed consent to participate in this study.

## Author contributions

WG: Data curation, Formal analysis, Investigation, Methodology, Writing – original draft. FL: Data curation, Formal analysis, Methodology, Writing – original draft. LF: Data curation, Formal analysis, Methodology, Writing – original draft. ZD: Data curation, Formal analysis, Methodology, Writing – original draft. XZ: Data curation, Formal analysis, Methodology, Writing – original draft. WC: Data curation, Formal analysis, Methodology, Writing – original draft. YY: Conceptualization, Data curation, Formal analysis, Funding acquisition, Investigation, Methodology, Project administration, Resources, Software, Supervision, Validation, Visualization, Writing – original draft, Writing – review & editing. YW: Conceptualization, Data curation, Formal analysis, Funding acquisition, Investigation, Methodology, Project administration, Resources, Software, Supervision, Validation, Visualization, Writing – original draft, Writing – review & editing.
