# Supplementary material for: Genetic mechanisms underlying local spontaneous brain activity in episodic migraine
Source: Front Neurosci. 2024 Feb 6;18:1348591. doi: 10.3389/fnins.2024.1348591 (PMC10876778; doi:10.3389/fnins.2024.1348591)
Supplement: Supplementary file 1 [file Data_Sheet_1.docx]

**Supplementary Materials**

| **Donor** | **Age (years)** | **Gender** | **Ethnicity** | **Hemisphere** | **Post-mortem interval (h)** |
| --- | --- | --- | --- | --- | --- |
| H0351.2001 | 24 | Male | African American | Both | 23 |
| H0351.2002 | 39 | Male | African American | Both | 10 |
| H0351.1009 | 57 | Male | Caucasian | Left | 25.5 |
| H0351.1012 | 31 | Male | Caucasian | Left | 17.5 |
| H0351.1015 | 49 | Female | Hispanic | Left | 30 |
| H0351.1016 | 55 | Male | Caucasian | Left | 18 |

**Table S1. Demographic information of the six adult donors in the AHBA**

Abbreviations: AHBA, Allen Human Brain Atlas.

| Clusters | Peak MNI coordinates (x, y, and z) | Number of voxels | Max T value |
| --- | --- | --- | --- |
| Two-sample *t*-test  (EM > HCs) |  |  |  |
| Rectus_L | -6, 33, -24 | 126 | 4.8476 |

**Table S2.** **Brain areas with significant differences in the ALFF values between EM patients and HCs (*P* < 0.05, cluster-level FWE-corrected)**

Abbreviations: ALFF, amplitude of low-frequency fluctuations; MNI, Montreal Neurological Institute; EM, episodic migraine; HCs, healthy controls; L, left.

**Table S3.** **Brain areas with significant differences in the fALFF values between EM patients and HCs (*P* < 0.05, cluster-level FWE-corrected)**

| Clusters | Peak MNI coordinates (x, y, and z) | Number of voxels | Max T value |
| --- | --- | --- | --- |
| Two-sample *t*-test  (EM > HCs) |  |  |  |
| Rectus_L | 9, 18, -27 | 37 | 4.8082 |
| Temporal_Mid_R | 42, -60, 0 | 41 | -4.8179 |
| Cingulum_Ant_L | 0, 27, 9 | 50 | 4.3746 |
| Occipital_Sup_R | 18, -90, 21 | 82 | -4.3269 |

Abbreviations: fALFF, fractional amplitude of low-frequency fluctuations; MNI, Montreal Neurological Institute; L, left; EM, episodic migraine; HCs, healthy controls; L, left; R, right.

**Table S4.** Cross-sample (n = 845) Pearson’s correlations between expression values of the genes and resting-state brain alterations (ReHo, ALFF and fALFF) (*P* < 0.05/5013 = 9.974 ×10^−6^)

|  | **ReHo** | **ALFF** | **fALFF** |
| --- | --- | --- | --- |
| ASTN2 | *r* = -0.203 | *r* = -0.266 | *r* = -0.179 |
| ATP2B1 | *r* = -0.187 | *r* = -0.231 | *r* = -0.194 |
| CACNA1A | *r* = -0.219 | *r* = -0.257 | *r* = -0.245 |
| CAMTA1 | *r* = -0.244 | *r* = -0.293 | / |
| INPP5A | *r* = -0.228 | *r* = -0.197 | *r* = -0.284 |
| INPP5B | / | *r* = -0.198 | / |
| LRCH1 | *r* = -0.242 | *r* = -0.368 | *r* = -0.263 |
| MEF2D | *r* = -0.172 | *r* = -0.272 | *r* = -0.172 |
| MLXIPL | *r* = 0.176 | *r* = 0.257 | *r* = 0.174 |
| NCOR2 | *r* = 0.233 | *r* = 0.315 | *r* = 0.183 |
| NFIB | *r* = -0.170 | / | *r* = -0.152 |
| PDZRN4 | *r* = 0.166 | *r* = 0.305 | / |
| PLA2G4A | / | *r* = 0.182 | / |
| PLCE1 | / | *r* = 0.164 | / |
| SLC24A3 | / | / | *r* = 0.172 |
| SNX24 | *r* = -0.166 | / | *r* = -0.204 |
| TBC1D16 | *r* = 0.190 | *r* = 0.192 | / |
| YAP1 | *r* = -0.237 | *r* = -0.233 | *r* = -0.257 |
| ZMYND8 | *r* = -0.156 | *r* = -0.237 | *r* = -0.170 |
| ZNF462 | *r* = 0.182 | *r* = 0.268 | *r* = 0.179 |
